# C57BL/6 and 129 inbred mouse strains differ in Gbp2 and Gbp2b expression in response to inflammatory stimuli *in vivo*

**DOI:** 10.12688/wellcomeopenres.15329.1

**Published:** 2019-08-20

**Authors:** Barbara Clough, Ryan Finethy, Rabia T. Khan, Daniel Fisch, Sarah Jordan, Harshil Patel, Jörn Coers, Eva-Maria Frickel

**Affiliations:** 1 Host-Toxoplasma Interaction Laboratory, The Francis Crick Institute, London, NW1 1AT, UK; 2 Department of Molecular Genetics and Microbiology, Duke University Medical Center, Durham, North Carolina, USA; 3 Bioinformatics and Biostatistics, The Francis Crick Institute, London, NW1 1AT, UK; 4 Department of Immunology, Duke University Medical Center, Durham, North Carolina, USA

**Keywords:** Guanylate binding proteins, Toxoplasma gondii, Shigella flexneri, Host-pathogen interaction, innate immune sensing

## Abstract

**Background**: Infections cause the production of inflammatory cytokines such as Interferon gamma (IFNγ). IFNγ in turn prompts the upregulation of a range of host defence proteins including members of the family of guanylate binding proteins (Gbps). In humans and mice alike, GBPs restrict the intracellular replication of invasive microbes and promote inflammation. To study the physiological functions of Gbp family members, the most commonly chosen *in vivo* models are mice harbouring loss-of-function mutations in either individual *Gbp *genes or the entire *Gbp *gene cluster on mouse chromosome 3. Individual *Gbp *deletion strains differ in their design, as some strains exist on a pure C57BL/6 genetic background, while other strains contain a 129-derived genetic interval encompassing the *Gbp *gene cluster on an otherwise C57BL/6 genetic background.

**Methods**: To determine whether the presence of 129 alleles of paralogous *Gbps *could influence the phenotypes of 129-congenic *Gbp*-deficient strains, we studied the expression of Gbps in both C57BL/6J and 129/Sv mice following *in vivo* stimulation with adjuvants and after infection with either *Toxoplasma* *gondii* or *Shigella flexneri*.

**Results**: We show that C57BL/6J relative to 129/Sv mice display moderately elevated expression of Gbp2, but more prominently, are also defective for Gbp2b (formerly Gbp1) mRNA induction upon immune priming. Notably, *Toxoplasma* infections induce robust Gbp2b protein expression in both strains of mice, suggestive of a *Toxoplasma*-activated mechanism driving Gbp2b protein translation. We further find that the higher expression of Gbp2b mRNA in 129/Sv mice correlates with a gene duplication event at the *Gbp2b* locus resulting in two copies of the *Gbp2b *gene on the haploid genome of the 129/Sv strain.

**Conclusions**: Our findings demonstrate functional differences between 129 and C57BL/6 *Gbp *alleles which need to be considered in the design and interpretation of studies utilizing mouse models, particularly for phenotypes influenced by Gbp2 or Gbp2b expression.

## List of Symbols and Abbreviations

CNV           Copy number variation

Gbp             Guanylate Binding Protein

PAMP          Pathogen-associated molecular pattern

IFNγ            interferon gamma

IP                intraperitoneally

LPS             lipopolysaccharide

CpG           unmethylated CpG DNA

Poly(I:C)     Polyinosinic-polycytidylic acid

## Introduction

Interferon gamma (IFNγ) production during an infection is important to control pathogen replication and mediate an effective host response. IFNγ regulates the expression of a multitude of genes, which includes genes encoding dynamin-like GTPases families: the Mx proteins, the very large interferon-inducible GTPases, the p47 immunity related GTPases (IRGs), and the p65 guanylate binding proteins (Gbps) ( Pilla-Moffett *et al*., 2016). The family of Gbp proteins is highly expressed upon IFNγ stimulation as well as following infections, for example with the protozoan *Toxoplasma gondii* or the bacterium *Listeria monocytogenes* ( Degrandi *et al*., 2007). Various Gbps have been shown to control *in vivo* murine infections with intracellular pathogens, such as BCG *Mycobacterium bovis* ( Kim *et al*., 2011) *Legionella pneumophila* ( Liu *et al*., 2018) and *Toxoplasma* ( Selleck *et al*., 2013). More recently, the family of Gbps has been shown to be involved in rapid activation of murine inflammasomes ( Finethy *et al*., 2015; Man *et al*., 2015; Meunier *et al*., 2014; Meunier *et al*., 2015; Pilla *et al*., 2014; Shenoy *et al*., 2012). Human GBPs have additionally been demonstrated to be important in the control of infection and host cell death. GBP2 and GBP5 inhibit zika virus, measles, influenza A and HIV infectivity ( Braun *et al*., 2019; Krapp *et al*., 2016), GBP1 acts on dengue virus, vesicular stomatitis virus and encephalomyocarditis virus ( Anderson *et al*., 1999; Pan *et al*., 2012) and GBP3 on influenza virus ( Nordmann *et al*., 2012). GBP1 impacts *Chlamydia trachomatis* replication inside macrophages ( Al-Zeer *et al*., 2013), controls intracellular growth of *Toxoplasma* ( Johnston *et al*., 2016) and blocks intracytosolic actin motility by *Shigella flexneri* ( Piro *et al*., 2017; Wandel *et al*., 2017). In terms of host cell death, human GBP5 was reported to promote NLRP3-dependent inflammasome activation in response to bacteria and soluble stimuli ( Shenoy *et al*., 2012) and GBP1 in macrophages promotes pyroptosis during *Salmonella* infection and apoptosis during *Toxoplasma* infection ( Fisch *et al*., 2019).

While the cell-intrinsic function of Gbps can be assessed in cell culture models, the interrogation of their physiological functions requires the use of *in vivo* mouse models, including *Gbp* gene deletion strains. Recently reported knockouts in *Gbp2* ( Finethy *et al*., 2017) and in *Gbp5* ( Meunier *et al*., 2014) used homologous recombination in C57BL/6-derived embryonic stem cells or zinc finger nuclease-based gene editing technology in C57BL/6 zygotes, respectively. However, previously reported *Gbp2b* (formerly *Gbp1*), *Gbp2* and *Gbp5* deletion strains were generated in 129-derived embryonic stem cells, and then backcrossed for multiple generations to C57BL/6 mice ( Degrandi *et al*., 2013; Kim *et al*., 2011; Shenoy *et al*., 2012), effectively generating congenic mice bearing an interval of 129 DNA surrounding the respective *Gbp* knockout loci.

 Previous work by Staeheli *et al*, has shown that a number of classical inbred mouse strains do not express Gbp2b protein in the spleen, upon stimulation *in vivo* with the pathogen-associated molecular pattern (PAMP) poly(I:C) ( Staeheli *et al*., 1984). The work stratified classical inbred mouse strains as “responders” [A/J, BALB/cJ, C3H/HeJ, 129/Ola] or “non-responders” [CBA/J, DBA/2J, C57BL/6J]. Further to this, Nguyen *et al*. have shown that the *Gbp2b* transcript is not detectable in the lung of C57BL/6J mice following intravenous injection with the PAMP lipopolysaccharide (LPS) ( Nguyen *et al*., 2002). Degrandi *et al*. confirmed that *in vitro* stimulation with poly(I:C) and LPS does not upregulate Gbp2b transcripts in C57BL/6J-derived ANA-1 macrophages, yet the study detected induced Gbp2b transcripts following *in vitro* stimulation with IFNγ ( Degrandi *et al*., 2007). *In vivo*, the induction of Gbp2b was also observed, both at a transcript and protein level, in C57BL/6J mice following infection with *L*. *monocytogenes* or *Toxoplasma* ( Degrandi *et al*., 2007). IFNγ-stimulated 129xC57BL/6J primary mouse embryonic fibroblasts (MEFs) presented with more Gbp2b protein compared to pure C57BL/6J MEFs as assessed by mass spectrometry ( Encheva *et al*., 2018). Thus, it is apparent that different mouse inbred genetic backgrounds vary in their ability to upregulate Gbp2b in response to different PAMP stimuli. However, despite the reported variation in *Gbp2b* gene expression, no previous study has systematically analysed the effect of different PAMPs on Gbp2b expression in “responder” and “non-responder” mice nor compared the genetic architecture of the *Gbp2b* gene across these two categories of mouse strains.

In this study, we examined the *Gbp2b* and *Gbp2* loci in the “non-responder” C57BL/6J and the “responder” 129/Sv mouse strains ( Staeheli *et al*., 1984). We determined the expression profile of Gbp2b and Gbp2 following *in vivo* stimulation with various PAMPs as well as systemic infections with either the protozoan pathogen *Toxoplasma* or the bacterial pathogen *S. flexneri*. We found that PAMP stimulation alone is sufficient to induce Gbp2b expression in 129/Sv but not in C57BL/6J mice, thus confirming and expanding observations made previously by Staeheli and colleagues ( Staeheli *et al*., 1984). Similarly, we found that infections with *S. flexneri* induced robust Gbp2b expression in 129/Sv but not C57BL/6J mice. In contrast to stimulation with individual purified PAMPs or *S. flexneri* infections, we unexpectedly found that infection with live *Toxoplasma* induced robust Gbp2b protein expression without any notable change in Gbp2b mRNA expression in “non-responder” C57BL/6J mice, suggesting that Gbp2b expression is regulated post-transcriptionally by *Toxoplasma*. Lastly, we also observed notably lower PAMP- or infection-induced expression of Gbp2 in 129/Sv compared to C57BL/6 mice. In conclusion, our studies reveal substantial, mouse strain-dependent variation in Gbp2b and Gbp2 expression. These findings need to be taken into consideration for the design and interpretation of *in vivo* mouse experiments employed for the study of GBP-related immune functions.

## Results

### *In vivo* administration of various PAMPs leads to robust Gbp2b expression in 129/Sv but not C57BL/6J mice

**Figure 1.**
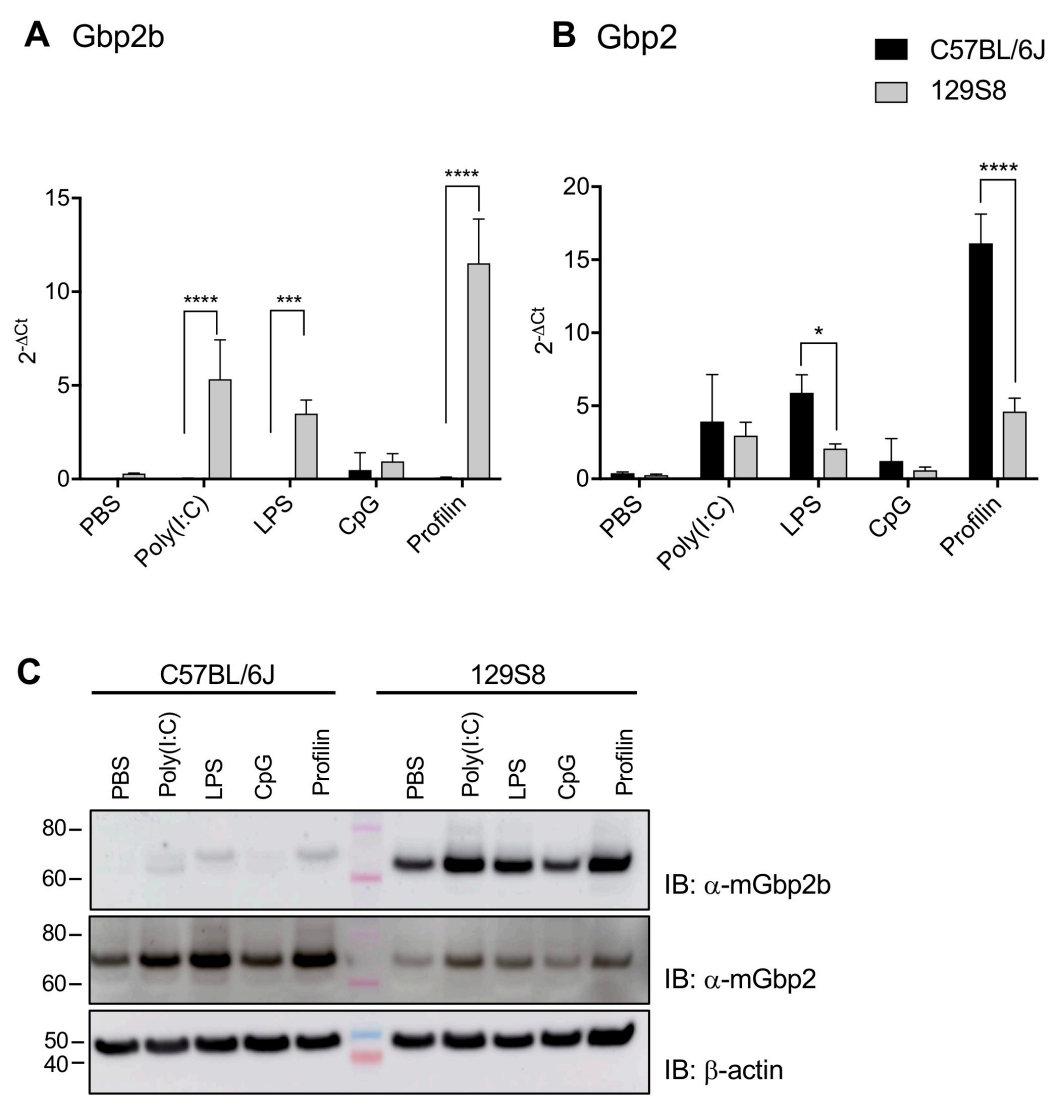
Expression of *Gbp2b* and *Gbp2* following various pathogen-associated molecular pattern (PAMP) injections. **A** and **B**) mRNA expression of Gbp2b ( **A**) and Gbp2 ( **B**), 6 hours post-intraperitoneal injection of various PAMPs. Analysis of whole spleens of C57BL/6J and 129/Sv mice. Data are represented as fold change over Hprt (2 ^-ΔCt^). Representative experiment with ≥ 3 mice/condition of n=3 experiments. 2-way ANOVA, ****, p<0.0001; ***, p<0.001. **C**) Immunoblot showing expression of Gbp2b and Gbp2 in protein lysates from spleens of C57BL/6J and 129/Sv mice. Spleens were taken 6 h after IP injection with various PAMPs. Representative immunoblot of n=2 experiments, β-actin as loading control.

A previous publication reported a lack of Gbp2b expression following immune stimulation with the TLR3/ RIG-I agonist polyinosinic-polycytidylic acid (poly(I:C)) in C57BL/6J mice *in vivo,* and thus proposed that C57BL/6J mice carry a *Gbp2b* loss-of-function allele ( Staeheli *et al*., 1984). Given the central role of GBPs in the innate immune response and the broad use of C57BL/6J mice in immunological research, we decided to systematically revisit these observations and to monitor the expression of Gbp2b mRNA and protein both in C57BL/6J and 129/Sv mice in response to various PAMPs. We initially analysed the expression of Gbp2b, as well as Gbp2 as a control reference transcript, in the spleens of mice 6h after injection with PBS (control), poly(I:C), lipopolysaccharide (LPS), unmethylated CpG DNA (CpG) or the *Toxoplasma* actin-binding protein profilin in order to stimulate RIG-I/TLR3, TLR4, TLR9 or TLR11/12, respectively. In contrast to 129/Sv mice, in which we detected robust induction of Gbp2b mRNA expression in response to poly(I:C), LPS and profilin, we observed no significant differences in Gbp2b expression following PAMP stimulation as compared to PBS in C57BL/6 mice ( Figure 1A, underlying data ( Clough *et al*., 2019)). While these findings confirmed previous observations ( Staeheli *et al*., 1984), we also noted diminished expression of Gbp2 mRNA in 129/Sv mice compared with C57BL/6J stimulated with either LPS or profilin ( Figure 1B, underlying data ( Clough *et al*., 2019)). These findings prompted us to monitor the expression of seven additional Gbp paralogs (Gbp3 – Gbp9). We observed that in contrast to the remarkable strain-dependent variation in Gbp2b and Gbp2 expression, mRNA expression of other Gbps was comparable in PAMP-stimulated 129/Sv and C57BL/6 mice. The sole exceptions to this were higher expression of Gbp7 and Gbp9 in 129/Sv mice injected with poly(I:C) and higher Gbp5 expression following profilin injection also in 129/Sv (Extended data Figure S1 ( Clough *et al*., 2019)). Analysis of protein levels of Gbp2b and Gbp2 after PAMP stimulation largely reflected the results obtained by transcriptional analysis: we found Gbp2b protein expression in the spleens of C57/BL6J mice to be at the threshold or below the level of detection, thus substantially diminished compared to Gbp2b protein expression in 129/Sv mice ( Figure 1C, underlying data ( Clough *et al*., 2019)). Also, in agreement with our mRNA expression data ( Figure 1B), we observed diminished protein expression of Gbp2 in 129/Sv spleens compared to the same tissue harvested from immune stimulated C57BL/6J mice ( Figure 1C, underlying data ( Clough *et al*., 2019)).

### *In vivo* infection with *Toxoplasma* induces marked Gbp2b protein expression in both mouse strains

**Figure 2.**
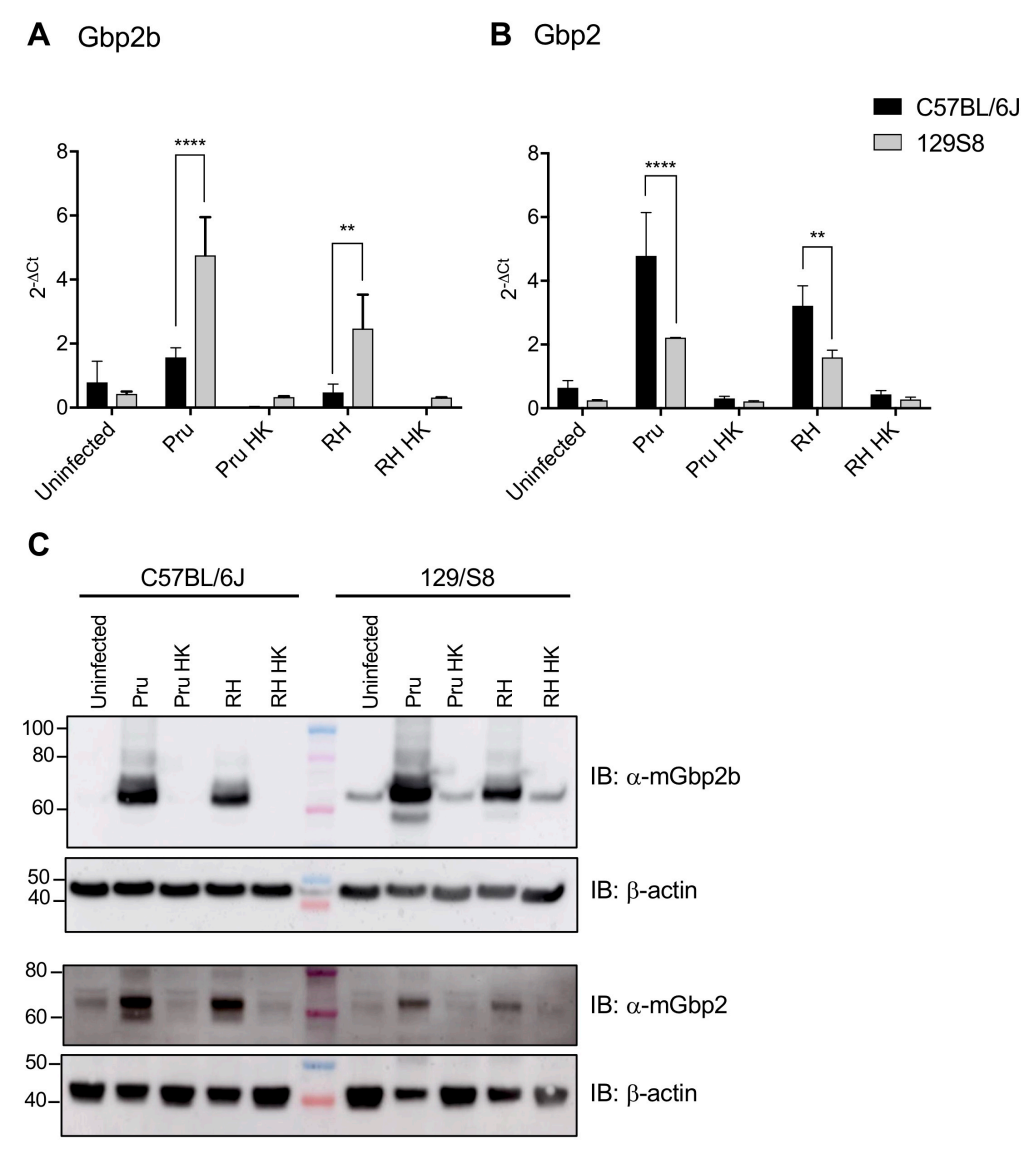
Expression of *Gbp2b* and *Gbp2* following *Toxoplasma gondii* infection. **A** and **B**) mRNA expression Gbp2b and Gbp2 was studied on day 8 in spleens of mice IP injected with live or heat killed (HK) *Toxoplasma* tachyzoites (20,000 for strain Pru, and 100 for strain RH). Data are represented as fold change over Hprt (2 ^-ΔCt^). Representative experiment with ≥ 3 mice/condition of n=3 experiments. 2-way ANOVA, ****, p<0.0001; **, p<0.01. **C**) Immunoblot showing expression of Gbp2b and Gbp2 in protein lysates from spleens of C57BL/6J and 129/Sv mice. Spleens were taken 8 days after mice were injected IP with *Toxoplasma* as described for **A** and **B**. Representative immunoblot of n=2 experiments, β-actin as loading control.

We next examined whether strain-dependent variation in Gbp2b and Gbp2 expression also occurred in infected animals. Confirming previous observations by Degrandi *et al*. ( Degrandi *et al*., 2007), we found the induction of Gbp2b and Gbp2 mRNA expression in the spleens of mice at 8 days post infection to occur only with live *Toxoplasma* but not with heat-killed (HK) parasites ( Figure 2A and B, underlying data ( Clough *et al*., 2019)). We additionally observed significantly higher expression of Gbp2b mRNA in the spleens of 129/Sv than in the spleens of C57BL/6J mice, infected with either type II (Pru) (p value < 0.0001) or type I (RH) (p value < 0.05) *Toxoplasma*, thus establishing that both PAMPs and *Toxoplasma* infections induce significantly more Gbp2b mRNA expression in 129/Sv than in C57BL/6J mice. Similarly, in agreement with our analysis of PAMP-triggered expression ( Figure 1), we found that Gbp2 mRNA expression was higher in C57BL/6J compared to 129/Sv mice following infection with either type II (Pru) or type I (RH) *Toxoplasma* ( Figure 2B). All other Gbps were expressed equally between C57BL/6J and 129/Sv with the exception of slightly lower levels of Gbp9 during type I (RH) infection (Extended data Figure S2 ( Clough *et al*., 2019)). We next analysed protein expression in the same animals and observed protein expression levels for Gbp2 as well as Gbp2b were prominently induced in the spleens of both C57BL/6J and 129/Sv mice after infection with live *Toxoplasma* ( Figure 2C, underlying data ( Clough *et al*., 2019)), in spite of an apparent lack of Gbp2b mRNA induction in *Toxoplasma*-infected C57BL6/J mice.

### *In vivo* infection with *S. flexneri* leads to robust Gbp2b expression in 129/Sv but not C57BL/6J mice

**Figure 3.**
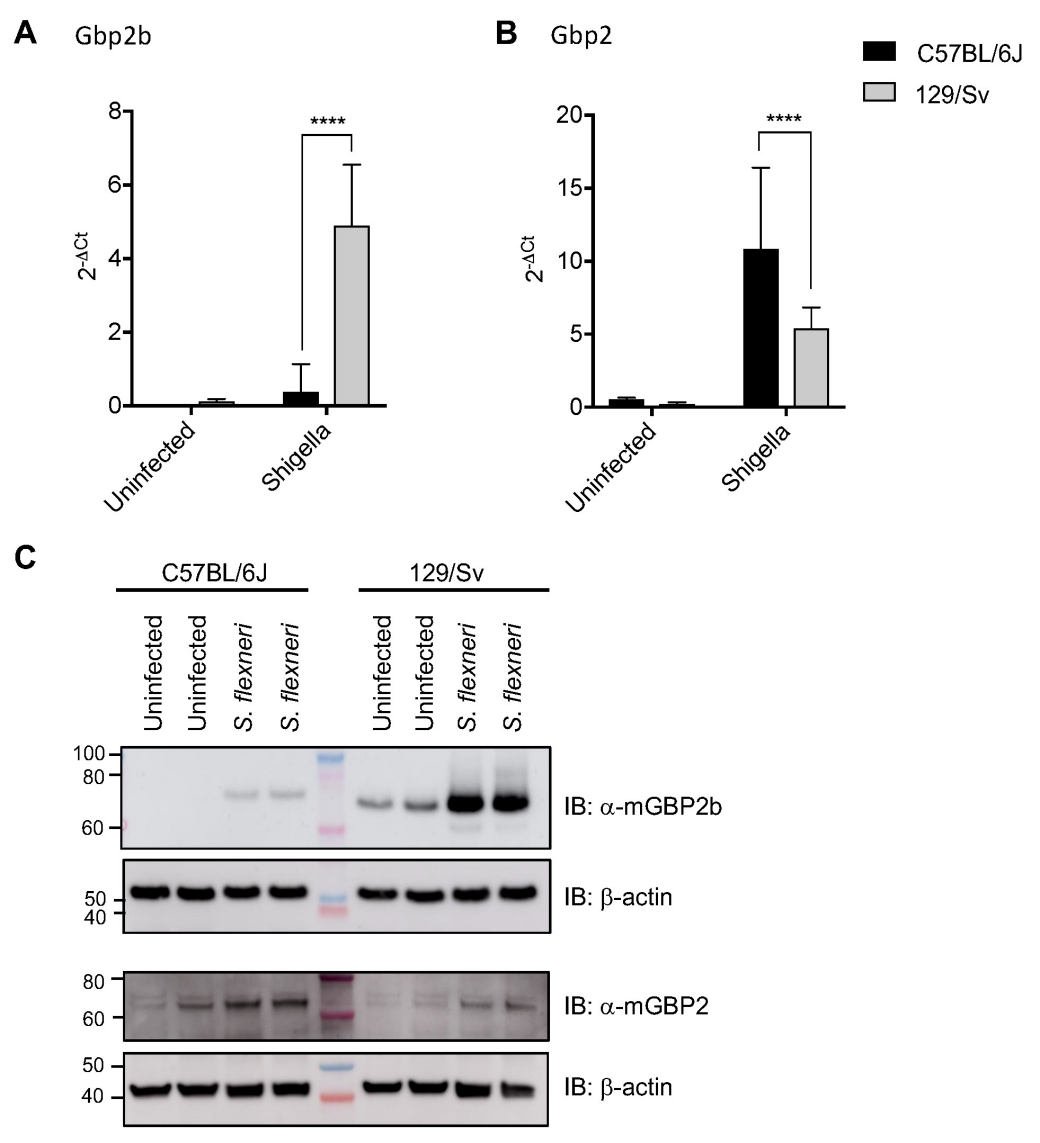
Expression of *Gbp2b* and *Gbp2* following *Shigella flexneri* infection (qPCR and IB). **A** and **B**) mRNA expression of Gbp2b and Gbp2 was studied 18 h p.i. in spleens of mice IP injected with *Shigella flexneri*. Data are represented as fold change over Hprt (2 ^-ΔCt^). Uninfected mice (n = 3/strain); infected C57BL/6J (n = 15) and 129/S8 (n = 16). **C**) Immunoblot showing expression of Gbp2b and Gbp2 in protein lysates from spleens of C57BL/6J and 129/Sv mice. Spleens were taken 18h after injection IP with *S. flexneri*. Representative immunoblot of two mice each of the cohort described in **A** and **B**, β-actin as loading control.

In contrast to the administration of various PAMPs *in vivo* ( Figure 1), we had found that infection with live *Toxoplasma* induced substantial Gbp2b expression in ‘non-responder’ C57BL/6J mice ( Figure 2). This led us to question whether a second infectious agent could similarly provide an induction signal for Gbp2b expression that the PAMP administration alone was lacking. Based on a recent study demonstrating a role for Gbps in resistance to *S. flexneri* infections in mice ( Li *et al*., 2017), we intraperitoneally infected C57BL/6J and 129/Sv mice with *S. flexneri* serotype 2a and monitored mRNA and protein expression at 18 hours-post-infection (hpi). Mirroring our observations with LPS and other PAMPs ( Figure 1), we recorded a more than 10-fold induction of Gbp2b mRNA in 129/Sv mice, while expression of Gbp2b was only minimally induced in C57BL/6J mice ( Figure 3A, underlying data ( Clough *et al*., 2019)). As seen with injected PAMPs ( Figure 1B), we observed an inverse relationship for Gbp2 mRNA expression, which was significantly reduced in 129/Sv compared to C57BL/6J mice ( Figure 3B, underlying data ( Clough *et al*., 2019)). All other Gbps were expressed to similar levels during *S. flexneri* infection (Extended data Figure S3 ( Clough *et al*., 2019)). These strain-dependent differences in mRNA expression correlated with corresponding differences in protein expression, where we observed more robust expression of Gbp2b protein in 129/Sv than in C57BL/6J mice, while expression levels of Gbp2 protein were moderately reduced in 129/Sv mice ( Figure 3C, underlying data ( Clough *et al*., 2019)). These results demonstrated that both purified PAMPs as well as *S. flexneri* infections lead to high expression of Gbp2b mRNA and protein, but relatively low expression of Gbp2 in 129/Sv compared to C57BL/6J mice.

### The 129/Sv genome contains a 25 kb gene duplication spanning *Gbp2b* and *Gbp2*

**Figure 4.**
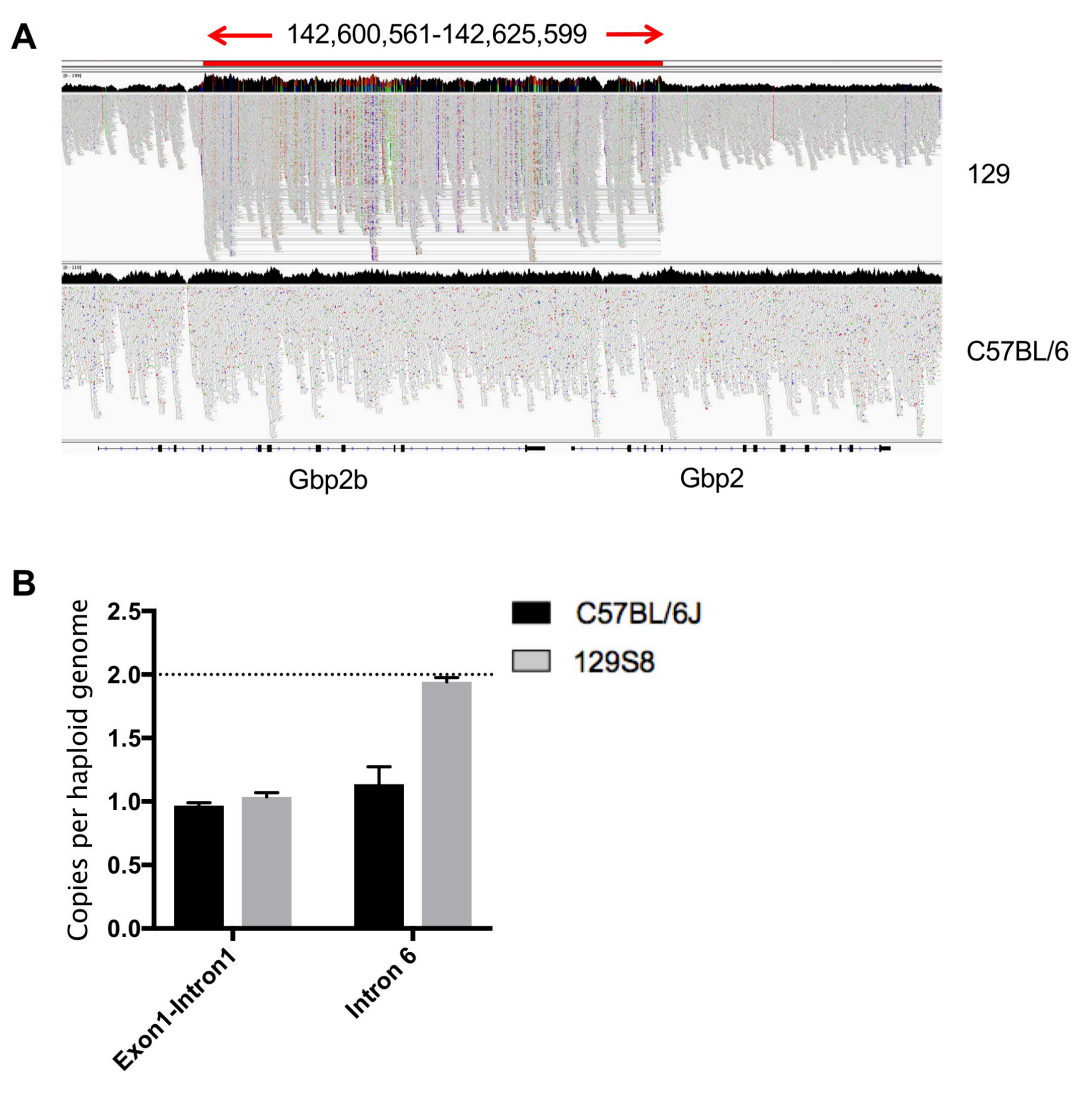
Copy number variation within the *Gbp2b* in inbred mouse strains. **A**) Integrative genomics viewer (IGV) screenshot confirming the presence of a copy number gain (chr3:142600561-142625599) in 129S1/SvImJ (top-panel; coverage = 199) relative to C57BL/6NJ (bottom-panel; coverage = 110) overlapping the *Gbp1* and *Gbp2* locii. Read alignment files were obtained from the Mouse Genomes Project ( ftp://ftp-mouse.sanger.ac.uk/current_bams). **B**) Digital PCR using primers located on two distinct exons was used to assess the presence of CNV on the C57BL/6J and 129/Sv genetic background. Representative of two experiments using 3 mice each. The 129/Sv has only one copy of at exon 1 (two on the diploid genome), but two copies of exon 6 (four on the diploid genome).

Next, we set out to identify any SNPs or structural variants that could explain the differences in expression of Gbp2b observed in the C57BL/6J and 129/Sv murine strains (Extended data Supplementary Table 1 ( Clough *et al*., 2019)). We compared intronic, exonic and UTR (2kb upstream) sequences of *Gbp2b* in C57BL/6J and 129/Sv mice *.* We identified a small number of intronic, but no exonic SNPs that differ between C57BL6/J and 129/Sv (Extended data Supplementary Table 1 ( Clough *et al*., 2019)). Notably, our analysis additionally identified a copy number variation (CNV) in the *Gbp2b* gene locus ( Figure 4A, underlying data ( Clough *et al*., 2019)). This 25kb gene duplication event begins at exon 5 of *Gbp2b* and continues to exon 4 of *Gbp2* in the 129/Sv genome ( Figure 4A and Extended data Supplementary Table 2 ( Clough *et al*., 2019)).

Using qPCR we confirmed the presence of an insertion within the 129/Sv strain, that is not present in the C57BL/6J strain ( Figure 4B, underlying data ( Clough *et al*., 2019)). The probe located at the intron-exon boundary of exon 1 of *Gbp2b* confirmed that both C57BL/6J and 129/Sv only carry one copy on the haploid genome, similar to the reference. The CNV probe located in intron 6 confirmed the presence of two *Gbp2b* copies on the haploid genome of 129/Sv compared to one copy on the C57BL/6J genome ( Figure 4). These data demonstrate the presence of a CNV in the *Gbp2b* locus of 129/Sv beginning after exon 1 and extending into exon 6. Remarkably, the segregation pattern of this CNV ( Table 1) correlated with responder and non-responder phenotypes described previously ( Staeheli *et al*., 1984), suggesting a possible causative relationship.

**Table 1.** qPCR primer sequences (previously published ( Yamamoto *et al*., 2012)).

Gene		Sequence 5' → 3'
*Gbp2b*	fwd	ACCTGGAGACTTCACTGGCT
	rev	TTTATTCAGCTGGTCCTCCTGTATCC
*Gbp2*	fwd	CTGCACTATGTGACGGAGCTA
	rev	CGGAATCGTCTACCCCACTC
*Gbp3*	fwd	CTGACAGTAAATCTGGAAGCCAT
	rev	CCGTCCTGCAAGACGATTCA
*Gbp4*	fwd	GGAGAAGCTAACGAAGGAACAA
	rev	TTCCACAAGGGAATCACCATTTT
*Gbp5*	fwd	CTGAACTCAGATTTTGTGCAGGA
	rev	CATCGACATAAGTCAGCACCAG
*Gbp6*	fwd	AAGACCATGATATGATGCTGA
	rev	GAAAATCCATTTAAGAGAGCC
*Gbp7*	fwd	TCCTGTGTGCCTAGTGGAAAA
	rev	CAAGCGGTTCATCAAGTAGGAT
*Gbp8*	fwd	ACATCTGTCCATGAACCATGAAG
	rev	AAACCGTGATTCTGTCCTGCC
*Gbp9*	fwd	ACCGGGAATAGACTGGGTACT
	rev	CCGGGCCACACTTGTCATA
*Gbp10*	fwd	AAGACCATAACATGATGCTGA
	rev	GAAAATCCATTTAAGAGACA
*Gbp11*	fwd	GAAAGCTGAGGAAATGAGAAGAG
	rev	GCCTTTTCAATCAGTAAAGAGG
*Hprt*	fwd	TCAGTCAACGGGGGACATAAA
	rev	GGGGCTGTACTGCTTAACCAG

## Discussion

The GBP family consists of 11 members in mice and 7 in humans and has emerged as a critical regulator of antimicrobial host defence and inflammation ( Mitchell & Isberg, 2017; Pilla-Moffett *et al*., 2016; Saeij & Frickel, 2017; Tretina *et al*., 2019). Even though the importance of this protein family in antimicrobial immunity is now evident, the molecular function of individual GBP family members remains largely unexplored. To fill this gap in knowledge, several recent studies focused on the function of discrete human GBPs in cell-autonomous immunity ( Braun *et al*., 2019; Krapp *et al*., 2016; Li *et al*., 2017; Piro *et al*., 2017; Wandel *et al*., 2017). While these cell-based studies have provided several critical insights into the cellular functions of individual GBPs, we depend on animal models to detail the complex organismal responses orchestrated by these important immune proteins. Accordingly, the mouse, as the most widely used animal model for the study of inflammation and immunity, has been applied to dissect *Gbp* gene function *in vivo* ( Finethy *et al*., 2017; Kim *et al*., 2011; Meunier *et al*., 2014; Shenoy *et al*., 2012; Yamamoto *et al*., 2012). Occasionally, results from these mouse studies resulted in contradictory findings, as exemplified for mouse Gbp5 and its potential role in NLRP3 inflammasome activation ( Man *et al*., 2015; Meunier *et al*., 2014; Shenoy *et al*., 2012). It is difficult to untangle the exact nature of the reported differences, as macrophages employed in these studies were not treated uniformly. Nevertheless, one potential contributing factor to these discrepancies is the use of mouse strains with individual gene deletions, e.g. in *Gbp5*, that due to the process by which they were generated bear either C57BL/6 or 129 alleles of neighbouring *Gbp* paralogs. Thus, proper interpretation of results obtained with *Gbp* knockout strains requires an understanding of the differences between C57BL/6 and 129 *Gbp* alleles and their effects on gene expression and function.

Here, we analysed the expression profile of Gbp2b and Gbp2 following *in vivo* immune stimulation. We found that Gbp2b expression is vigorously induced in response to systemic immune activation by PAMPs or *S. flexneri* infection in 129/Sv, but not C57BL6/J mice, while Gbp2 expression was generally higher in C57BL6/J compared to 129/Sv mice. Higher induction of Gbp2b mRNA expression in 129/Sv mice correlated with a *Gbp2b* gene duplication event in the 129/Sv genome. Previous studies demonstrated an appreciable correlation between CNV and gene expression in the genome of different inbred mouse strains ( Chaignat *et al*., 2011; Henrichsen *et al*., 2009; Orozco *et al*., 2009), suggesting that the partial *Gbp2b* gene duplication extending into the *Gbp2* locus could cause the enhanced expression of Gbp2b and the reduced expression of Gbp2 in 129/Sv mice. CNV can impact gene expression through complex mechanisms that go beyond simple gene dosage effects ( Weischenfeldt *et al*., 2013). This is especially true when the CNV only partially overlaps with the complete gene segment, as is the case for the *Gbp2b/ Gbp2* duplication present in the 129/Sv genome. Thus, defining the molecular link between the *Gbp2b/ Gbp2* CNV and gene expression may prove to be difficult to ascertain. Nonetheless, considering the association of CNV with phenotypic variation ( Weischenfeldt *et al*., 2013), future studies focused on *GBP* gene variants need to not only evaluate disease association with *GBP* SNPs, as successfully demonstrated for the host response to viral infections ( Koltes *et al*., 2015), but also monitor the potential association of *GBP* CNVs with disease in animals and humans.

Whereas the delivery of PAMPs or infection with *S. flexneri* resulted in negligible induction of either Gbp2b mRNA or protein expression in C57BL/6J mice, infection with live but not heat-inactivated *Toxoplasma* led to robust Gbp2b protein expression in the absence of corresponding induction of Gbp2b mRNA expression in the same mouse strain. These data therefore suggest that infections with live *Toxoplasma* boost Gbp2b translation by an unknown mechanism. Future studies are needed to explore the nature of this mechanism, identify its molecular trigger and determine whether this response is induced by pathogens other than *Toxoplasma.* Here, we show that *S. flexneri* infections fail to deliver this putative molecular activator and accordingly *S. flexneri* infections drive robust Gbp2b expression predominantly or entirely through increased mRNA expression, a response present in 129/Sv, but absent from C57BL/6J mice. Considering the previously reported antimicrobial activities of Gbp2b ( Kim *et al*., 2011), we can therefore expect that the genetic origin of the *Gbp2b* allele, i.e. 129- versus C57BL/6-derived, will influence the outcome of infection studies with *Shigella* or related proteobacteria.

Because functional differences between 129 and C57BL/6 *Gbp* alleles in linkage with a given *Gbp* knockout allele are expected to affect host immune response and thus confound the interpretation of phenotypes associated with these *Gbp* knockout lines, future studies need to address these concerns. One strategy would be to generate congenic mouse lines bearing the 129-derived *Gbp* gene cluster on an otherwise C57BL/6 background and to use these mice as controls for *Gbp* knockout lines bearing similar 129 congenic DNA elements. While this strategy is admittedly burdensome, ignoring the impact of carrier 129 *Gbp* alleles on phenotypes associated with these *Gbp* knockout mice will inevitably lead to data misinterpretation and incorrect assignments of gene functions. The alternative strategy is to resort to the exclusive use of those *Gbp* knockout mouse lines that were generated in a pure C57BL/6 genetic background or to generate novel *Gbp* knockout alleles in any desired genetic background. With the advent of CRISPR-mediated genome editing technology, the production of individual *Gbp* knockout lines that are coisogenic with any given control strain has become remarkably trivial and thus renders the latter strategy highly feasible.

## Methods

### Ethics statement

All procedures involving mice were approved by the local ethical committee of the Francis Crick Institute Ltd, Mill Hill Laboratory and are part of a project license (PPL 80/2616) approved by the Home Office, UK, under the Animals (Scientific Procedures) Act 1986, or were approved by the Institutional Animal Care and Use Committees at Duke University (protocol registry number A113-16-05). Duke University maintains an animal program that is registered with the United States Department of Agriculture (USDA), assured through the National Institutes of Health/Public Health Service (NIH/PHS), and accredited with Association for Assessment and Accreditation of Laboratory Animal Care (AAALAC), International (accreditation number 363).

### Parasite culture

*Toxoplasma gondii* avirulent type II strain Pru and type I RH strain was used, both a gift from Jeroen Saeij. All strains of *Toxoplasma gondii* were maintained by serial passage on monolayers of HFF cells, cultured in DMEM with GlutaMAX (Life Technologies) supplemented with 10% FBS (Life Technologies), at 37°C in 5% CO _2_ ( Clough *et al*., 2016).

### Animal procedures

***In vivo Toxoplasma gondii infection***. Mice ( *Mus musculs*) C57BL/6J and 129/S8 were bred at the Francis Crick Institute Ltd. Animals were kept under specific pathogen free (SPF) conditions, housed in rodent facility in individually ventilated cages (GM500 from Tecniplast) (3–4 animals per cage) on standard Aspen bedding (Datesand, UK) with red mouse house enrichment. Animals were housed in light/dark cycle 12:12 (light on at 7am), temperature 19–23°C, Rh 45–65%. Commercial mouse diet (T2018S, Envigo, UK) and water available ad *libitum* via automated watering system (Edstrom). 6- to 8-week-old male mice (weight between 20–25g) were used. Animals were divided into experimental cohorts. Each cohort was assigned 3 mice and experiment was repeated 2–3 times to achieve statistical significance. In order to study the transcriptional and translational upregulation of murine Gbps after infection, mice were injected IP with live, or heat killed *Toxoplasma gondii*, with either 20,000 tachyzoites of the type II, avirulent Pru strain or 100 tachyzoites of the virulent type I RH strain. *Toxoplasma* gondii was heat killed by incubation at 65 **°**C for 20 minutes. Mice were euthanised by cervical dislocation in order to analyse the *in vivo* response to infection by harvesting spleens at 8 days after infection and analysed for *Gbp2b* and *Gbp2* expression. All efforts were made to ameliorate harm to the animals through careful statistical analysis employing the minimum amount of animals to achieve statistical significance.

***In vivo Shigella flexneri infection.*** Mice ( *Mus musculs*) C57BL/6J (The Jackson Laboratory, #000664) and 129SvEvTac (Taconic #129SVE) were bred at Duke University Medical Center. Animals were kept under SPF conditions, housed in rodent facility in Allentown IVC140 double sided racks/ Jag 75 cages (4 - 5 animals per cage) on 1/8 ^th^ of an inch standard corncob bedding (The Andersons lab bedding, USA). Animals were housed in light/dark cycle 12:12 (light on at 7am), temperature 20–23°C, Rh 30–70%. Commercial mouse diet (5053 diet, Purina, USA) and bottled tap water was available ad *libitum*. Wildtype *S. flexneri* serotype 2a (2457T) was grown overnight in tryptic soy broth (TSB) (Sigma, #22092) at 37°C with aerosolization. Saturated cultures were diluted 1:50 in 5 ml fresh TSB and incubated for 2.5 to 4 h at 37°C with shaking until absorbance of samples at a wavelength of 600 nm (OD _600_) reached 0.8 to 0.9, measured on a Smart-Spec 3000 (BioRad). Bacteria were washed and resuspended in PBS to a final concentration of 1 × 10 ^7^ colony forming units (CFUs) / mL. Eight male and eight female 129/Sv mice and age- and sex-matched C57BL/6J mice (6 – 12 week of age; weight between 16–27g) were grouped in 4 cohorts of 8 mice and *i.p.* injected with 5 × 10 ^6 ^CFUs. At 18 hpi mice were euthanized and spleens were harvested. Spleens were cut into two roughly equal portions, which were either processed for protein lysates or placed into TRIzol (15596026, Invitrogen) for RNA purification. Organs from uninfected control animals were processed in the same fashion.

***In vivo PAMP stimulation.*** Mice were injected IP with Profilin (0.5μg/100μl) (Sigma, SRP8050), Poly(I:C) (100μg/100μl) (Sigma, P1530), CpG (5μg/100μl) (Invivogen, tlrl-1826), LPS (100μg/100μl) (Invivogen, tlrl-3pelps) or PBS. Spleens were collected 6 hours after injection and analysed for *Gbp2b* and *Gbp2* expression.

***Quantitative polymerase chain reaction.*** Cellular RNA was extracted using the TRIzol (15596026, Invitrogen). RNA quality was determined on a Nanodrop 2000 Spectrophotometer (Thermo Scientific). RNA (2 μg) was reverse transcribed using the high-capacity cDNA synthesis kit (4368813, Applied Biosystems). qPCR used PowerUP SYBR green (A25742, Applied Biosystems) kit, 10 ng cDNA in a 10 μL reaction and primers (Sigma, see Table 1) at 1 μM final concentration on a QuantStudio 12K Flex Real-Time PCR System (Applied Biosystems). The standard PowerUP SYBR cycling program was used: UDG activation 50°C 2 minutes, Dual-Lock™ DNA polymerase 95°C 2 minutes, 40 cycles: Denature 95°C 15 seconds, Anneal/extend 60°C 1 minute. Recorded C _t_ values were normalised to the recorded C _t_ of murine *Hprt1* and data plotted as ΔC _t_ (Relative expression).

### Immunoblots

Protein lysates, prepared from mouse spleens, were run on SDS-PAGE (NuPage 4–12% Bis-Tris, ThermoFisher), immunoblotted and probed for expression of Gbp2b (Rabbit polyclonal antibody 1:5000, ( Virreira Winter *et al*., 2011)) or Gbp2 (goat anti-GBP2 1:200, Santa Cruz #sc-10588). Mouse monoclonal anti-α-actin antibody was used to control for protein loading (1:5000, Sigma #A5441). Secondary HRP-conjugated antibodies (goat anti-rabbit HRP ThermoFisher #G21234 1:20000; donkey anti-goat HRP Abcam #ab97110 1:5000, rabbit anti-mouse HRP Sigma #SAB3701023 1:20,000) were detected by chemiluminescence (Merck Millipore #WBKLS0500).

### Digital PCR

Digital PCR was used to assess the presence of copy number variation in the *Gbp2b* gene using a GeneAMP ^®^ PCR system 9700 (Applied Biosystems ^®^) and the QuantStudio™ 3D Digital PCR 20K Chip Kit v2 and Master Mix (Thermo Fisher, A26317). DNA extracted from tails of C57BL/6J and 129/Sv mice and a total concentration of 50ng was used for each chip. The 3D master-mix (Thermo Fisher, A26317), probes and DNA were prepared as per manufacturer’s instructions and 14.5 µL was loaded onto the chip. The two probes used were Mm00733848_Cn (intron 6) and Mm00095526_cn (overlaps exon 1 and intron 1). The probe TaqMan® Copy Number Reference Assay (Thermo Fisher, 4458369), Mouse, Tert, which has one copy on the mouse haploid genome was used as the reference. The chips (Thermo Fisher, A26317) were sealed and loaded onto the GeneAMP ^®^ PCR system 9700 (Applied Biosystems ^®^) and cycled according to the following parameters: 96 °C for 10 minutes, followed by 39 cycles of 60 °C for 2 min and 98 °C for 30 sec, and a final extension at 60 °C for 2 min. After cycling, the end-point fluorescence of the partitions on the chips was measured by transferring the chips to the measurement unit (Thermo Fisher, A26317). The data was analysed using the QuantStudio™ 3D Analysis Suite™ Software v3.0.

### Sequence analysis

Known SNPs and structural variations that are different between the 129/Sv and C57BL/6J were downloaded from Mouse phenome database (MPD; http://www.jax.org/phenome) and the whole genome sequencing data for the same strains were obtained from the Mouse Genomes Project.

### Statistical analysis

Graphs were plotted using Prism 8.0.2 (GraphPad Inc.) and presented as means of N = 3 experiments (with usually 3 technical repeats within each experiment) with error bars representing SEM, if not stated otherwise. Data analysis used two-way ANOVA.

## Data availability

### Underlying data

Figshare: Title: C57BL/6 and 129 inbred mouse strains differ in Gbp2 and Gbp2b expression in response to inflammatory stimuli *in vivo*.

**https://doi.org/10.6084/m9.figshare.8235524.v2** ( Clough *et al*., 2019)

This project contains the following underlying data:

- Fig1+S1_QPCR_PAMPs_2.pzfx (All raw data for Figure 1 and Extended data Supplementary Figure 1 in a Prism File.)
- Fig2+S2_QPCRToxo_1.pzfx (All raw data for Figure 1 and Extended data Supplementary Figure 1 in a Prism File.)
- Fig3+S3_Shigella-infection.pzfx (All raw data for Figure 1 and Extended data Supplementary Figure 1 in a Prism File.)
- Immunoblots.pptx (Uncropped immunoblots for Figure 1, Figure 2 and Figure 3 in a Powerpoint File.)
- Digital qPCR_Exp2 (All raw data for Figure 4 in a Prism File.)
- Fig1+S1_QPCR_PAMPs_GBP1.txt (All raw data for Figure 1 and Extended data Supplementary Figure 1 for GBP1 in TAB format.)
- Fig1+S1_QPCR_PAMPs_GBP2.txt (All raw data for Figure 1 and Extended data Supplementary Figure 1 for GBP2 in TAB format.)
- Fig1+S1_QPCR_PAMPs_GBP3.txt (All raw data for Figure 1 and Extended data Supplementary Figure 1 for GBP3 in TAB format.)
- Fig1+S1_QPCR_PAMPs_GBP4.txt (All raw data for Figure 1 and Extended data Supplementary Figure 1 for GBP4 in TAB format.)
- Fig1+S1_QPCR_PAMPs_GBP5.txt (All raw data for Figure 1 and Extended data Supplementary Figure 1 for GBP5 in TAB format.)
- Fig1+S1_QPCR_PAMPs_GBP6.txt (All raw data for Figure 1 and Extended data Supplementary Figure 1 for GBP6 in TAB format.)
- Fig1+S1_QPCR_PAMPs_GBP7.txt (All raw data for Figure 1 and Extended data Supplementary Figure 1 for GBP7 in TAB format.)
- Fig1+S1_QPCR_PAMPs_GBP8.txt (All raw data for Figure 1 and Extended data Supplementary Figure 1 for GBP8 in TAB format.)
- Fig1+S1_QPCR_PAMPs_GBP9.txt (All raw data for Figure 1 and Extended data Supplementary Figure 1 for GBP9 in TAB format.)
- Fig1+S1_QPCR_PAMPs_GBP11.txt (All raw data for Figure 1 and Extended data Supplementary Figure 1 for GBP11 in TAB format.)
- Fig2+S2_QPCRToxo_GBP1.txt (All raw data for Figure 2 and Extended data Supplementary Figure 2 for GBP1 in TAB format.)
- Fig2+S2_QPCRToxo_GBP2.txt (All raw data for Figure 2 and Extended data Supplementary Figure 2 for GBP2 in TAB format.)
- Fig2+S2_QPCRToxo_GBP3.txt (All raw data for Figure 2 and Extended data Supplementary Figure 2 for GBP3 in TAB format.)
- Fig2+S2_QPCRToxo_GBP4.txt (All raw data for Figure 2 and Extended data Supplementary Figure 2 for GB4 in TAB format.)
- Fig2+S2_QPCRToxo_GBP5.txt (All raw data for Figure 2 and Extended data Supplementary Figure 5 for GBP1 in TAB format.)
- Fig2+S2_QPCRToxo_GBP6.txt (All raw data for Figure 2 and Extended data Supplementary Figure 2 for GBP6 in TAB format.)
- Fig2+S2_QPCRToxo_GBP7.txt (All raw data for Figure 2 and Extended data Supplementary Figure 2 for GBP7 in TAB format.)
- Fig2+S2_QPCRToxo_GBP8.txt (All raw data for Figure 2 and Extended data Supplementary Figure 2 for GBP8 in TAB format.)
- Fig2+S2_QPCRToxo_GBP9.txt (All raw data for Figure 2 and Extended data Supplementary Figure 2 for GBP9 in TAB format.)
- Fig2+S2_QPCRToxo_GBP11.txt (All raw data for Figure 2 and Extended data Supplementary Figure 2 for GBP11 in TAB format.)
- Fig3+S3_Shigella-infection_Gbp1.txt (All raw data for Figure 3 and Extended data Supplementary Figure 3 for GBP1 in TAB format.)
- Fig3+S3_Shigella-infection_Gbp2.txt (All raw data for Figure 3 and Extended data Supplementary Figure 3 for GBP2 in TAB format.)
- Fig3+S3_Shigella-infection_Gbp3.txt (All raw data for Figure 3 and Extended data Supplementary Figure 3 for GBP3 in TAB format.)
- Fig3+S3_Shigella-infection_Gbp4.txt (All raw data for Figure 3 and Extended data Supplementary Figure 3 for GBP4 in TAB format.)
- Fig3+S3_Shigella-infection_Gbp5.txt (All raw data for Figure 3 and Extended data Supplementary Figure 3 for GBP5 in TAB format.)
- Fig3+S3_Shigella-infection_Gbp6.txt (All raw data for Figure 3 and Extended data Supplementary Figure 3 for GBP6 in TAB format.)
- Fig3+S3_Shigella-infection_Gbp7.txt (All raw data for Figure 3 and Extended data Supplementary Figure 3 for GBP7 in TAB format.)
- Fig3+S3_Shigella-infection_Gbp8.txt (All raw data for Figure 3 and Extended data Supplementary Figure 3 for GBP8 in TAB format.)
- Fig3+S3_Shigella-infection_Gbp9.txt (All raw data for Figure 3 and Extended data Supplementary Figure 3 for GBP9 in TAB format.)
- Fig3+S3_Shigella-infection_Gbp11.txt (All raw data for Figure 3 and Extended data Supplementary Figure 3 for GBP11 in TAB format.)

### Extended data

Figshare: Title: C57BL/6 and 129 inbred mouse strains differ in Gbp2 and Gbp2b expression in response to inflammatory stimuli *in vivo*.

**https://doi.org/10.6084/m9.figshare.8235524.v2** ( Clough *et al*., 2019)

This project contains the following Extended data:

- Gbp2b_ExtendedDataFigureS1.jpg (Extended data Figure S1 as a jpeg File.)
- Gbp2b_ExtendedDataFigureS2.jpg (Extended data Figure S2 as a jpeg File.)
- Gbp2b_ExtendedDataFigureS3.jpg (Extended data Figure S3 as a jpeg File.)
- Gbp2b_ExtendedDataSupplementaryTable 1.xlsx (Extended data Supplementary Table 1 as an Excel File.)
- Gbp2b_ExtendedDataSupplementaryTable 2.xlsx (Extended data Supplementary Table 2 as an Excel File.)

Data are available under the terms of the Creative Commons Attribution 4.0 International license (CC-BY 4.0).

Legends for Extended data:

**Extended data Figure S1. Expression of Gbp3-11 in C57BL/6J compared to the 129/Sv following IP injection with various PAMPs.** mRNA expression of Gbp3-9, 6 hours post-intraperitoneal injection of various PAMPs. Analysis of whole spleens of C57BL/6J and 129/Sv mice. Data are represented as fold change over HPRT (2 ^-ΔCt^). Representative experiment with ≥ 3 mice/condition of n=3 experiments. 2-way ANOVA, ****, p<0.0001; **, p<0.01.

**Extended data Figure S2. Expression of Gbp3-11 in C57BL/6J compared to the 129/Sv following *Toxoplasma gondii* infection.** mRNA expression of mouse Gbp3-9 was studied on day 8 in spleens of mice IP injected with live or heat killed (HK) *Toxoplasma* tachyzoites (20,000 for strain Pru, and 100 for strain RH). Data are represented as fold change over HPRT (2 ^-ΔCt^). Data are represented as fold change over HPRT (2 ^-ΔCt^). Representative experiment with ≥ 3 mice/condition of n=3 experiments. 2-way ANOVA, *, p=0.0286.

**Extended data Figure S3. Expression of Gbp3-11 in C57BL/6J compared to the 129/Sv following *Shigella flexneri* infection.** Data are represented as fold change over HPRT (2 ^-ΔCt^). Uninfected 6 mice/strain; infected 15 mice/C57BL/6 and 16 mice/129/S8.

**Extended data Supplementary Table 1. Single nucleotide polymorphisms of *Gbp2b* and *Gbp2* in 129 versus C57BL/6 mice.** Known SNPs and structural variations that are different between the 129/Sv and C57BL/6J were downloaded from Mouse phenome database (MPD; http://www.jax.org/phenome).

**Extended data Supplementary Table 2. Genetic variation of *Gbp2b* and *Gbp2* in 129 versus C57BL/6J mice.** Table summarizing the location of the gene duplication event within the context of the *Gbp2b* and *Gbp2* genes. The location of the dPCR and qPCR Taqman probes is also marked.

## Author information

Authors Barbara Clough, Ryan Finethy, Rabia T. Khan and Daniel Fisch contributed equally and share authorship amongst themselves. Authors Jörn Coers and Eva-Maria Frickel contributed equally and share authorship amongst themselves.
