# Supplementary material for: C57BL/6 and 129 inbred mouse strains differ in Gbp2 and Gbp2b expression in response to inflammatory stimuli in vivo
Source: Wellcome Open Res. 2019 Aug 20;4:124. [Version 1] doi: 10.12688/wellcomeopenres.15329.1 (PMC6749937; doi:10.12688/wellcomeopenres.15329.1)
Supplement: Peer review history

# Peer review: Reviewer response for version 1

**DOI:** 10.21956/wellcomeopenres.16734.r36269

**Published:** 2019-09-17

**Authors:** Igor Kramnik

**Affiliations:** 1 National Emerging Infectious Diseases Laboratory, Boston University School of Medicine, Boston, MA, USA

The study by Clough and co-workers demonstrates variation in the expression of two Gbp genes, Gbp2 and Gbp2b, between inbred mouse strains C57BL/6J and 129/S8. After stimulations with several PAMPs *in vivo*, the commonly used B6 mice expressed higher levels of Gbp2 and lower levels of Gbp2b mRNAs and proteins, as compared to the 129/S8 mice. Importantly, other Gbp genes encoded within the same locus were induced by PAMPs to a similar degree. It is well known that transferring a KO mutation to another background by backcrosses (from 129 to B6 in this case) results in transfer of an approximately 10 cM chromosome segment containing flanking genes. The authors correctly emphasize that the observed differences in Gbp2 and Gbp2b gene expression could influence the phenotypes of Gbp KO mice and lead to inconsistencies among the studies performed using Gbp gene knockouts created in the 129 and B6 backgrounds. To avoid the effect of polymorphisms within the flanking regions of the Gbp locus, the authors propose to use CRISPR for inactivating individual Gbp genes in a single background. However, their findings also suggest that the Gbp2b KO in the B6 mice may have less pronounced phenotype, as compared to 129 background, for example, just because this gene is less prominently expressed in the B6 genetic background and other Gbps may play compensatory roles. 

 The authors identified a genetic polymorphism within the mouse Gbp locus that is potentially responsible for the observed differences in the Gbp2 and Gbp2b genes expression: a duplication of a DNA segment overlapping these genes. Although it seems likely that this CNV is mechanistically linked to the observed interstrain differences in the Gbp2 and Gbp2b gene expression, this observation does not constitute a definitive proof. The strain distribution pattern (SDP) of the genetic polymorphism and the phenotype would strengthen this link. However, Table 1 does not contain the SDP data, as claimed in the text. This statement needs to be corrected. 

 Figures 1 and 3 demonstrate differential induction of Gbp2b mRNA 6 h after stimulation with PAMPs or 18 h after acute infection with *Shigella flexneri.* In both cases there is a good correlation between mRNA and protein levels. However, after 8 days of infection with *T.gondii*, the levels of the Gbp2b protein in B6 mice is significantly upregulated, while the mRNA levels are not. The authors propose an unknown mechanism of translational upregulation as an explanation, which does not seem convincing, however, since the mRNA levels in the B6 mice remain low, while the protein levels are clearly upregulated. Although this phenomenon is not central for this paper, it would be helpful to discuss alternative explanations as well. 

 Overall, this manuscript presents information relevant for studying the roles of Gbp proteins in host immunity using KO mice. This is a nice and clearly presented example of potential sources of inconsistencies between studies using gene knockouts due to background and “passenger gene” effects.

I confirm that I have read this submission and believe that I have an appropriate level of expertise to confirm that it is of an acceptable scientific standard.

# Peer review: Reviewer response for version 1

**DOI:** 10.21956/wellcomeopenres.16734.r36267

**Published:** 2019-08-23

**Authors:** Peter Staeheli

**Affiliations:** 1 Faculty of Medicine, University of Freiburg, Freiburg, Germany

This is a nice study that helps solving some confusion regarding the regulation of the *Gbp2* gene by IFN-γ and/or parasite infection in different mouse strains. Work published in 1984 had shown that inbred mouse strains B6 and 129 differ with regard to *Gbp2* regulation, but later work with knockout mice yielded conflicting results. The new work finds a convincing explanation for these discrepancies. It turns out that knockout mice generated from ES cells derived from 129 mice continue to carry a 129-like *Gbp2b* locus even after extensive backcrossing to B6 mice. Accordingly, the important take-home message of this manuscript is that one should be extremely careful when drawing conclusions from results with *Gbp* knockout mice which were produced using traditional technology that relied on ES cells from 129 mice.  

 The paper is well written. There are only a few minor issues that should be addressed:

1. The statement in the introduction that IFN-γ induces dynamin-like GTPases, including Mx proteins is not correct. *Mx* genes are exceptional in that they are induced by type I and type III IFN, but not by IFN-γ.
2. The authors use the term “IFNγ” as short form for interferon-γ. According to current terminology rules, the correct abbreviation is “IFN-γ”.
3. In the last paragraph of the results section it is mentioned that Table 1 shows how markers for exon 1 and exon 6 of the *Gbp2* gene segregate in different mouse strains. However, Table 1 is showing qPCR primer sequences. The missing information should be provided.

I confirm that I have read this submission and believe that I have an appropriate level of expertise to confirm that it is of an acceptable scientific standard.
